# Efficacy of immune checkpoint inhibitors plus molecular targeted agents after the progression of lenvatinib for advanced hepatocellular carcinoma

**DOI:** 10.3389/fimmu.2022.1052937

**Published:** 2022-12-09

**Authors:** Fucun Xie, Bowen Chen, Xu Yang, Huaiyuan Wang, Ge Zhang, Yanyu Wang, Yunchao Wang, Nan Zhang, Jingnan Xue, Junyu Long, Yiran Li, Huishan Sun, Ziyu Xun, Kai Liu, Xiangqi Chen, Yang Song, Xiaobo Yang, Zhenhui Lu, Yilei Mao, Xinting Sang, Yinying Lu, Haitao Zhao

**Affiliations:** ^1^ Department of Liver Surgery, State Key Laboratory of Complex Severe and Rare Diseases, Peking Union Medical College Hospital, Chinese Academy of Medical Sciences and Peking Union Medical College, Beijing, China; ^2^ Department of Thoracic Surgery, National Cancer Center/National Clinical Research Center for Cancer/Cancer Hospital, Chinese Academy of Medical Sciences and Peking Union Medical College, Beijing, China; ^3^ State Key Laboratory of Molecular Oncology, National Cancer Center/National Clinical Research Center for Cancer/Cancer Hospital, Chinese Academy of Medical Sciences and Peking Union Medical College, Beijing, China; ^4^ Central Laboratory, National Cancer Center/National Clinical Research Center for Cancer/Cancer Hospital & Shenzhen Hospital, Chinese Academy of Medical Sciences and Peking Union Medical College, Shenzhen, China; ^5^ Peking University 302 Clinical Medical School, Beijing, China; ^6^ Department of Thoracic Surgery, State Key Laboratory of Complex Severe and Rare Diseases, Peking Union Medical College Hospital, Chinese Academy of Medical Sciences and Peking Union Medical College, Beijing, China; ^7^ Hepatobiliary and Pancreatic Surgery, Shenzhen Qianhai Shekou Free Trade Zone Hospital, Shenzhen, China

**Keywords:** hepatocellular carcinoma (HCC), immune checkpoint inhibitor (ICI), molecular targeted agent (MTA), lenvatinib, efficacy, safety

## Abstract

**Background:**

Lenvatinib is a standard first-line systemic therapy in advanced hepatocellular carcinoma (aHCC) and is widely used in all lines. However, the efficacy and safety of immune checkpoint inhibitors (ICIs) plus molecular targeted agents (MTAs) after the progression of lenvatinib treatment are unclear.

**Objective:**

The aim of this study was to evaluate the anticancer effects of ICI plus MTA in patients with aHCC who progressed after lenvatinib.

**Methods:**

We retrospectively included aHCC patients treated with ICI plus MTA after the progression of lenvatinib from two medical centers. Participants who continued lenvatinib treatment were classified into the “ICI+Lenva” group, while the “ICI+Others” group included patients receiving other MTAs. The efficacy endpoints were progression-free survival (PFS), post-progression survival (PPS), overall survival (OS), and tumor response following RECIST v1.1. Safety was evaluated according to Common Terminology Criteria for Adverse Events v5.0.

**Results:**

In this study, 85 eligible aHCC patients were enrolled, including 58 in the ICI+Lenva group and 27 in the ICI+Others group. At a median follow-up time of 22.8 months, the median PPS and PFS were 14.0 (95% CI: 9.0-18.2) and 4.5 months (95% CI: 3.5-8.3), respectively. The objective response and disease control rates were 10.6% and 52.9%, respectively. No significant differences were observed in any of the efficacy endpoints between the two groups. Prolonged PPS was associated with Child–Pugh grade A, AFP < 400 IU/ml, and concomitant locoregional treatment. All patients experienced adverse events (AEs), but no fatal AEs were observed.

**Conclusion:**

ICI plus MTA in aHCC patients after the progression of lenvatinib presented high antitumor activity and safety. Patients could continue lenvatinib treatment and receive ICIs as well as locoregional treatment to achieve better OS.

## Introduction

Hepatocellular carcinoma (HCC) is a highly malignant carcinoma with a dismal prognosis, especially advanced HCC (aHCC) (1). Lenvatinib, a molecular targeted agent (MTA), showed promising results compared with sorafenib in the REFLECT clinical trial (2), which made it the second MTA approved by the Food and Drug Administration as first-line systemic therapy for aHCC following sorafenib. A real-world study confirmed that lenvatinib performed well as a post-line systemic therapy in aHCC patients (3).

Immunotherapy is currently making progress in improving the survival of aHCC. Immune checkpoint inhibitors (ICIs) contribute to overcoming immune evasions by targeting immune checkpoints, including programmed cell death protein 1 (PD-1), programmed cell death ligand 1 (PD-L1), and cytotoxic T lymphocyte-associated antigen-4 (CTLA-4) (4). Anti-PD-1 inhibitor monotherapy and MTA plus ICIs have exhibited specific antitumor activity in aHCC (5–10).

However, not all aHCC patients respond to lenvatinib in real-world clinical practice. Moreover, resistance and progression were observed in a large portion of aHCC patients treated with lenvatinib. Multiple systemic treatment regimens have been brought into clinical practice for HCC patients after the progression of first-line sorafenib treatment. Regorafenib was the first MTA approved for second-line treatment, followed by cabozantinib and ramucirumab (11–13). Pembrolizumab and camrelizumab also displayed satisfying prognoses as non-first-line treatments (5, 6, 9).

Recent studies have pointed out that antitumor treatments after progression with first-line lenvatinib were associated with prolonged survival in aHCC patients (14, 15). However, the optimal subsequent therapy after the progression of lenvatinib was inconclusive partially because it has been only four years since the approval of lenvatinib as a first-line agent for aHCC patients. A previous study reported that regorafenib plus PD-1 inhibitor was a promising treatment pattern after the progression of first-line sorafenib or lenvatinib (16), which indicated that the ICI plus MTA pattern (17–19) might be a promising strategy for aHCC patients with progression of lenvatinib treatment. In addition, many patients continued to use lenvatinib after progression due to Patient Assistance Programs, but the efficacy of rechallenging lenvatinib with lenvatinib plus ICI remains questionable. In this study, we aimed to explore the effectiveness and safety of MTAs plus ICIs and the necessity of MTA rotation in aHCC patients after the progression of lenvatinib treatment.

## Materials and methods

### Study design

This study was a multicenter observational retrospective real-world study that included two medical centers (Peking Union Medical College Hospital, PUMCH; The Fifth Medical Centre of PLA General Hospital, PLAGH). Informed consent was not obtained from the study participants, as this was a retrospective study. The requirement for informed consent was waived by the Ethics Committees of PUMCH and PLAGH, as specific patient details are not presented here (JS-1391 and KY-2022-8-68-1). All data were collected from the electronic medical records system (EMRS). This study conformed to the Declaration of Helsinki and was approved by the Ethics Committee mentioned above. The study is registered at ClinicalTrials.gov (NCT03892577).

### Patients and groups

Eligible patients were ICI-therapy-naïve aHCC patients who received MTA plus ICI directly after radiology-confirmed progression of lenvatinib treatment under RECIST v1.1 in two medical centers from Jan 2018 to Dec 2021. Clinicians made therapy decisions for each participant after a comprehensive evaluation of stage, liver function, physical performance, the patient’s preference, and Chinese clinical guidelines for managing hepatocellular carcinoma (20).

The major inclusion criteria were as follows: [1] at least one measurable tumor region according to RECIST v1.1; [2] received at least one regimen of MTA plus ICI directly after the progression of lenvatinib treatment; and [3] at least one effective follow-up. The major exclusion criteria were as follows: [1] end-stage HCC (defined as BCLC stage D); [2] did not suffer from progression of lenvatinib treatment or discontinued lenvatinib treatment due to other reasons instead of disease progression; [3] received other systemic therapies after progression of lenvatinib treatment; [4] without any effective follow-up; and [5] had already received ICI therapy before the progression of lenvatinib treatment.

The patients underwent an examination every six to eight weeks. According to the different patterns of combination therapy, patients who rechallenged lenvatinib plus ICI were included in the “ICI+Lenva” group, while those who switched to another MTA plus ICI were included in the “ICI+Others” group.

### Endpoints and follow-up

The primary endpoint was post-progression survival (PPS, defined as the time between the initiation of MTA plus ICI after the progression of lenvatinib and death, the last-time effective survival follow-up or the end of the study, whichever came first). In particular, we calculated overall survival (OS), which started from the initiation of lenvatinib treatment to the finishing point mentioned above. The secondary endpoints were progression-free survival (PFS, defined as the time between the initiation of MTA plus ICI after the progression of lenvatinib and radiology-confirmed disease progression, death, or the last radiologic evaluation, whichever came first), objective response rate (ORR), and disease control rate (DCR). Patients with complete response (CR), partial response (PR), or stable disease (SD) ≥ six months continuously were defined as achieving a clinical benefit response (CBR). All secondary efficacy endpoints were evaluated according to RECIST v1.1. Safety was evaluated with the Common Terminology Criteria for Adverse Events (CTCAE) v5.0.

### Risk factors for prognosis and subgroup analysis

Cox proportional hazards analyses were employed to explore the risk factors for PPS and PFS. Furthermore, to rule out the effect of previous systemic therapy before lenvatinib treatment on efficacy, we extracted patients treated with first-line lenvatinib for subgroup analysis.

### Statistical analysis

Baseline characteristics, ORR, DCR, and CBR are described as numbers (ratio, %) and were compared with Fisher’s exact test. Survival analyses were performed with the Kaplan–Meier method, and differences were compared with the log-rank test. A Cox proportional hazards model was used to analyze risk factors for prognosis. Variables with P < 0.05 in univariate analysis were included in multivariate analysis. The significance level was defined as a two-tailed P < 0.05. All statistical analyses were conducted with R v3.6.3.

## Results

### Baseline characteristics

In our study, 85 eligible patients were continually enrolled, 63 from PUMCH and 22 from PLAGH. Among these 85 participants, 58 were in the ICI+Lenva group, and the remaining 27 were in the ICI+Others group. In the ICI+others group, 14 patients switched to apatinib, six to regorafenib, four to bevacizumab, two to sorafenib, and one to donafenib. PD-1 inhibitors were applied in 82 patients (96.5%), while the other three patients (3.5%) used PD-L1 inhibitors. Previous systemic therapies were reported in 30 (35.3%) patients before lenvatinib treatment. In addition, 21 (24.7%) patients received regional therapies during the period of ICI plus MTA therapy. Detailed baseline characteristics are recorded in **Table 1**. No significant differences were observed between the ICI+Lenva group and the ICI+Others group.

**Table 1:** Baseline characteristics of patients.

Characteristics	All patients N = 85, N (%)	ICI+Lenva N = 58, N (%)	ICI+Others N = 27, N (%)	P - value
Medical center				0.183
PUMCH	63 (74.1)	40 (69.0)	23 (85.2)	
PLAGH	22 (25.9)	18 (31.0)	4 (14.8)	
Age				1
< 65	67 (78.8)	46 (79.3)	21 (77.8)	
≥ 65	18 (21.2)	12 (20.7)	6 (22.2)	
Sex				0.508
Female	12 (14.1)	7 (12.1)	5 (18.5)	
Male	73 (85.9)	51 (87.9)	22 (81.5)	
Viral Hepatitis				0.804
HBV	66 (77.7)	46 (79.3)	20 (74.1)	
HCV	3 (3.5)	2 (3.5)	1 (3.7)	
NBNC	16 (18.8)	10 (17.2)	6 (22.2)	
Alcohol consumption				0.791
No	63 (74.1)	42 (72.4)	21 (77.8)	
Yes	22 (25.9)	16 (27.6)	6 (22.2)	
ECOG				0.35
0	32 (37.7)	23 (39.7)	9 (33.3)	
1	46 (54.1)	32 (55.2)	14 (51.9)	
2	7 (8.2)	3 (5.2)	4 (14.8)	
Child–Pugh				1
A	61 (71.8)	42 (72.4)	19 (70.4)	
B	24 (28.2)	16 (27.6)	8 (29.6)	
AFP (ng/ml)				1
< 400	53 (62.3)	36 (62.1)	17 (63.0)	
≥ 400	32 (37.7)	22 (37.9)	10 (37.0)	
Tumor number				0.660
1	6 (7.1)	5 (8.6)	1 (3.7)	
≥ 2	79 (92.9)	53 (91.4)	26 (96.3)	
Tumor size				0.625
< 5 cm	28 (32.9)	18 (31.0)	10 (37.0)	
≥ 5 cm	57 (67.1)	40 (69.0)	17 (63.0)	
Macrovascular invasion				0.168
No	47 (55.3)	29 (50.0)	18 (66.7)	
Yes	38 (44.7)	29 (50.0)	9 (33.3)	
Extrahepatic metastasis				0.81
No	31 (36.5)	22 (37.9)	9 (33.3)	
Yes	54 (63.5)	36 (62.1)	18 (66.7)	
BCLC staging				1
B	9 (10.6)	6 (10.3)	3 (11.1)	
C	76 (89.4)	52 (89.7)	24 (88.9)	
Previous systemic treatment				0.477
No	55 (64.7)	39 (67.2)	16 (59.3)	
Yes	30 (35.3)	19 (32.8)	11 (40.7)	
Previous locoregional treatment				1
No	12 (14.1)	8 (13.8)	4 (14.8)	
Yes	73 (85.9)	50 (86.2)	23 (85.2)	

PUMCH, Peking Union Medical College Hospital; PLAGH, Chinese Peoples’ Liberation Army General Hospital; HBV, hepatitis B virus; HCV, hepatitis C virus; NBNC, no HBV or HCV infection; AFP, alpha-fetoprotein; BCLC, Barcelona Clinic Liver Cancer.

### Overall efficacy

At the data cutoff (2022-05-15), progressive disease was observed in 61 patients treated with ICI plus MTA following lenvatinib progression. The major reason for combination therapy termination was disease progression or death (n = 54). The other seven cases were associated with adverse events (AEs) (**Table S1**). Only 19 patients switched to other ICI plus MTA treatment (n = 9) or MTA monotherapy (n = 10) after combination therapy progression, while the other 42 patients did not receive systemic therapy (**Table S2**). Patients active in post-line systemic therapy after progression of ICI plus MTA were likely to have prolonged OS (median OS 6.0 months vs. 20.3 months, P = 0.012, **Figure S1**).

**Figure 1:**
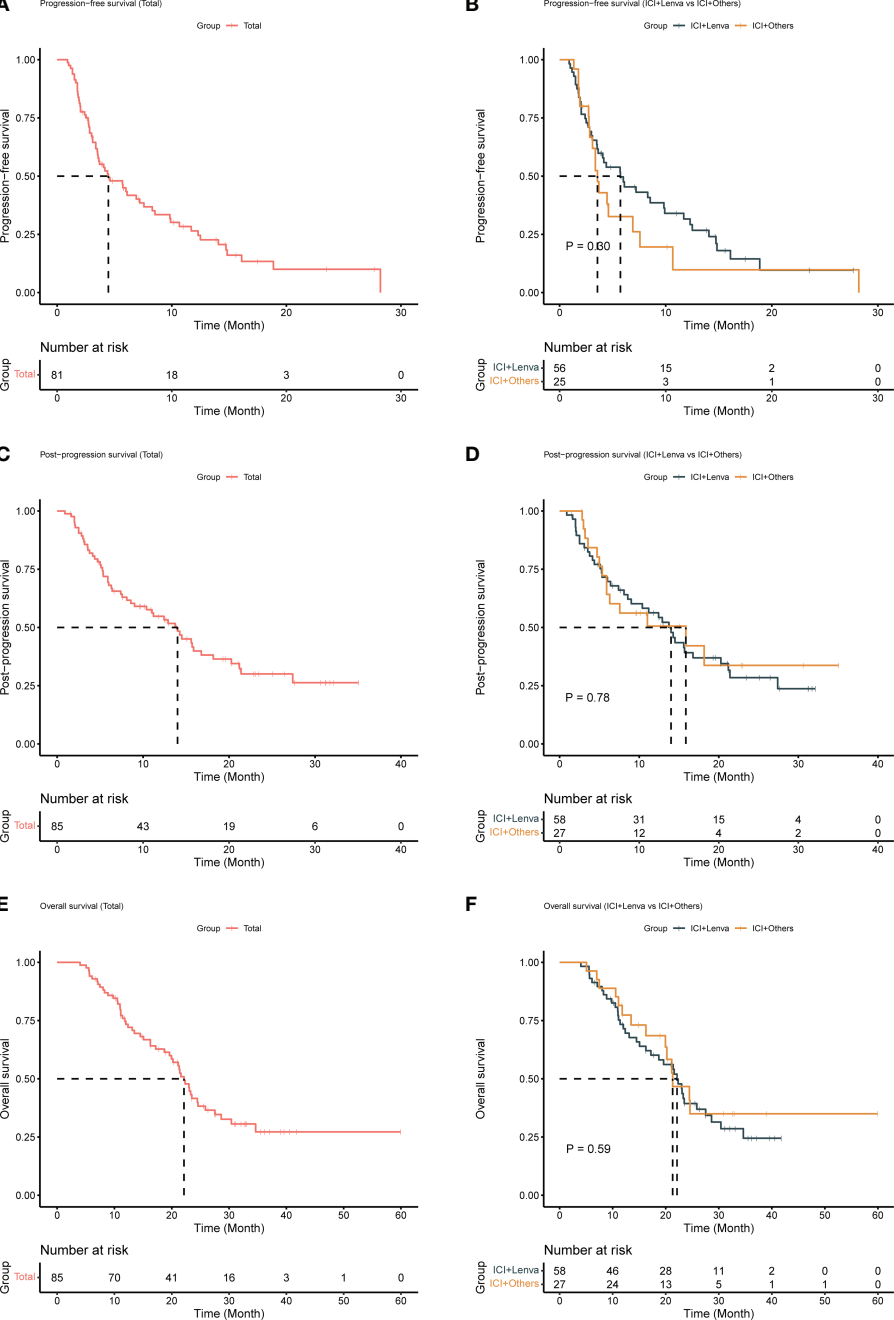
Survival outcomes for patients treated with ICI plus MTA after lenvatinib progression. **(A)** Progression-free survival for all patients; **(B)** Progression-free survival for the ICI+Lenva group and the ICI+Others group; **(C)** Post-progression survival for all patients; **(D)** Post-progression survival for the ICI+Lenva group and the ICI+Others group; **(E)** Overall survival for all patients; **(F)** Overall survival for the ICI+Lenva group and the ICI+Others group.

The median follow-up was 22.8 (95% CI: 19.6-27.6) months. The median PPS and PFS were 14.0 (95% CI: 9.0-18.2) months and 4.5 (95% CI: 3.5-8.3) months, respectively. The overall median OS was 22.1 (95% CI: 19.9-25.9) months. The overall ORR, DCR and CBR were 10.6% (95% CI: 5.0%-19.2%), 52.9% (95% CI: 41.8%-63.9%) and 32.9% (95% CI: 23.1%-44.0%), respectively (**Table 2** and **Figure 1**).

**Table 2:** Tumor response and efficacy endpoints for all patients.

Characteristics	All patients N = 85	ICI+Lenva N = 58	ICI+Others N = 27	P value
CR, N (%)	0 (0)	0 (0)	0 (0)	
PR, N (%)	9 (10.6)	8 (13.8)	1 (3.7)	
SD, N (%)	36 (42.4)	22 (37.9)	14 (51.9)	
PD, N (%)	36 (42.4)	26 (44.8)	10 (37.0)	
NE, N (%)	4 (4.7)	2 (3.5)	2 (7.4)	
**ORR (95% CI)**	10.6% (5.0-19.2)	13.8% (6.2-25.4)	3.7% (0.1-19.0)	0.304^a^
**DCR (95% CI)**	52.9% (41.8-63.9)	51.7% (38.2-65.1)	55.6% (35.3-74.5)	0.742
**CBR (95% CI)**	32.9% (23.1-44.0)	37.9% (25.5-51.6)	22.2% (8.6-42.3)	0.15
mPFS, (Months, 95% CI)	4.5 (3.5-8.3)	5.7 (3.6-11.7)	3.6 (3.1-NE)	0.3
mPPS, (Months, 95% CI)	14.0 (9.0-18.2)	14.0 (9.0-21.2)	15.9 (5.9-NE)	0.78
mOS, (Months, 95% CI)	22.1 (19.9-25.9)	22.1 (17.2-28.6)	21.3 (19.9-NE)	0.14

CR, complete response; PR, partial response; SD, stable disease; PD, progressive disease; NE, not evaluable; ORR, objective response rate; DCR, disease control rate; CBR, clinical benefit rate; PFS, progression-free survival; PPS, post-progression survival; OS, overall survival.

^a^Pearson’s chi-square test using continuity correction.

### Differences in the efficacy endpoints

Following the median follow-up times of 23.5 months and 15.1 months in the ICI+Lenva and ICI+Others groups, the median PPS and PFS were 14.0 (95% CI: 9.0-21.2) and 15.9 (95% CI: 5.9-NE) months (P = 0.78) and 5.7 (95% CI: 3.6-11.7) and 3.6 (3.1-NE) months (P = 0.3), respectively, with no significant differences. The ORR, DCR and CBR in the ICI+Lenva vs. ICI+Others groups were 13.8% (95% CI: 6.2%-25.4%) vs. 3.7% (95% CI: 0.1%-19.0%) (P = 0.304), 51.7% (95% CI: 38.2%-65.1%) vs. 55.5% (95% CI: 35.3%-74.5%) (P = 0.74), and 37.9% (95% CI: 25.5%-51.6%) vs. 22.2% (95% CI: 8.6%-42.2%) (P = 0.15), respectively. Additionally, the median OS was 22.1 (95% CI: 17.2-28.6) and 21.3 (95% CI: 19.9-NE) in the two respective groups (P = 0.14) (**Table 2** and **Figure 1**).

### Subgroup efficacy analysis

**Figure 2:**
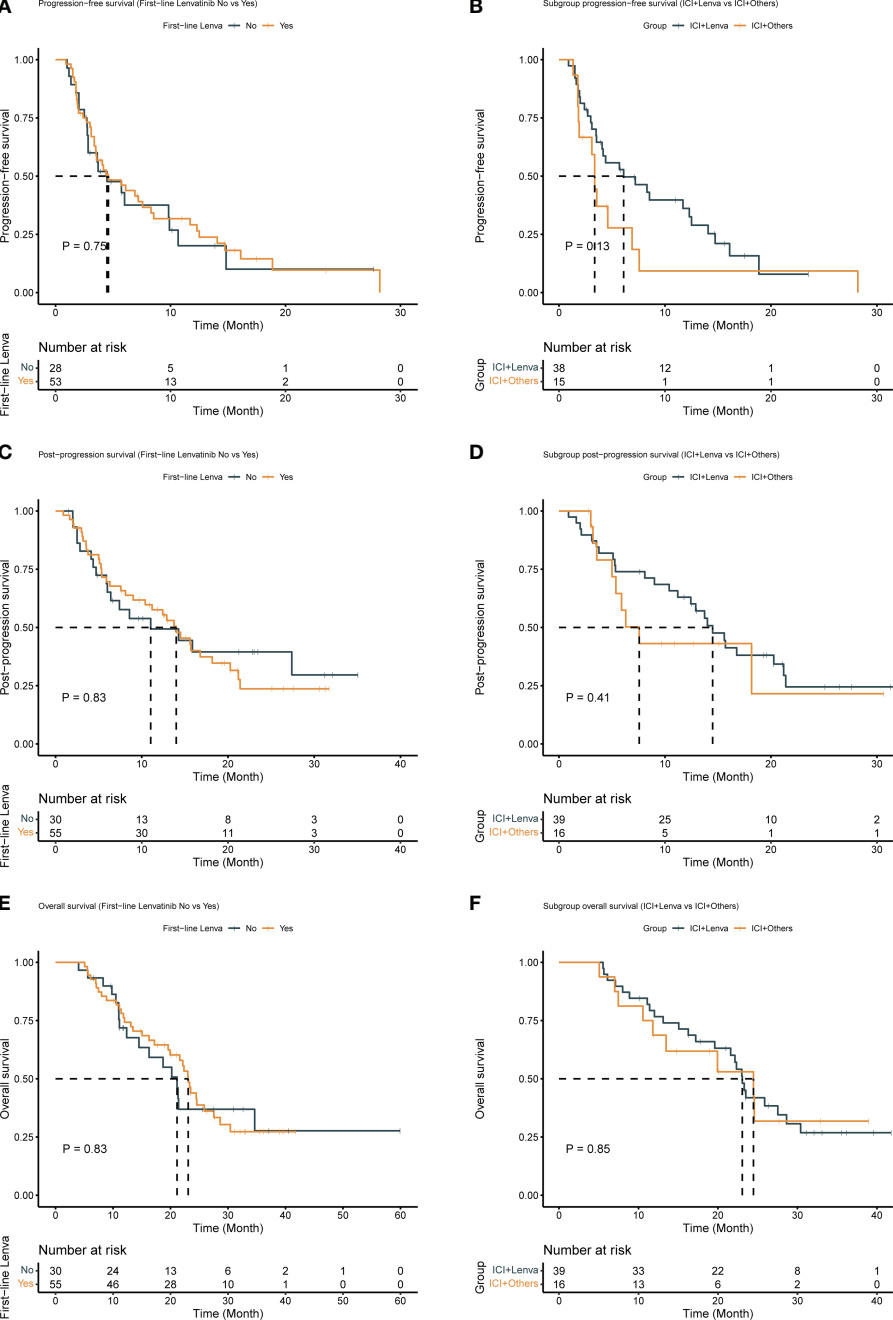
Survival outcomes for patients treated with ICI plus MTA after first-line lenvatinib progression. **(A)** Progression-free survival for patients after first-line or post-line lenvatinib progression; **(B)** Progression-free survival for the ICI+Lenva group and the ICI+Others group in first-line lenvatinib subgroup; **(C)** Post-progression survival for patients after first-line or post-line lenvatinib progression; **(D)** Post-progression survival for the ICI+Lenva group and the ICI+Others group in first-line lenvatinib subgroup; **(E)** Overall survival for patients after first-line or post-line lenvatinib progression; **(F)** Overall survival for the ICI+Lenva group and the ICI+Others group in first-line lenvatinib subgroup.

Subgroup analyses were performed in patients with first-line lenvatinib treatment (N = 55). The ORR, DCR, CBR, PFS, PPS, and OS in this subgroup were similar to those in the post-line lenvatinib group (**Figure 2**). The ORR, DCR and CBR in the ICI+Lenva group vs. the ICI+Others group were 18.0% (95% CI: 7.5-33.5) vs. 0 (95% CI: 0.0-20.6), 53.9% (95% CI: 37.2-69.9) vs. 37.5% (95% CI: 15.2-64.6), and 43.6% (95% CI: 27.8-60.4) vs. 18.8% (95% CI: 4.1-45.7), respectively. The median PFS, PPS and OS were 6.1 (4.1-14.1) vs. 3.4 (1.9 -NE), 14.5 (11.2-21.4) vs. 7.6 (5.4-NE), and 23.1 (19.9-28.6) vs. 23.1 (11.8-NE), respectively. Except for CBR, which showed a marginally significant difference, no significant differences were observed among the other efficacy endpoints in the subgroups (**Table 3** and **Figure 2**).

**Table 3:** Tumor response and efficacy endpoints for first-line lenvatinib subgroup analysis.

Characteristics	All patients N = 55	ICI+Lenva N = 39	ICI+Others N = 16	P value
CR, N (%)	0 (0)	0 (0)	0 (0)	
PR, N (%)	7 (12.7)	7 (18.0)	0 (0.0)	
SD, N (%)	20 (36.4)	14 (35.9)	6 (37.5)	
PD, N (%)	26 (47.3)	17 (43.6)	9 (56.3)	
NE, N (%)	2 (3.6)	1 (2.6)	1 (6.3)	
**ORR (95% CI)**	12.7% (5.3-24.5)	18.0% (7.5-33.5)	0 (0.0-20.6)	0.171^a^
**DCR (95% CI)**	49.1% (35.4-62.9)	53.9% (37.2-69.9)	37.5% (15.2-64.6)	0.271
**CBR (95% CI)**	36.4% (23.8-50.4)	43.6% (27.8-60.4)	18.8% (4.1-45.7)	0.082
mPFS, (Months, 95% CI)	4.6 (3.5-8.5)	6.1 (4.1-14.1)	3.4 (1.9 -NE)	0.13
mPPS, (Months, 95% CI)	14.0 (9.0-20.3)	14.5 (11.2-21.4)	7.6 (5.4-NE)	0.59
mOS, (Months, 95% CI)	22.1 (19.9-25.9)	23.1 (19.9-28.6)	23.1 (11.8-NE)	0.85

CR, complete response; PR, partial response; SD, stable disease; PD, progressive disease; NE, not evaluable; ORR, objective response rate; DCR, disease control rate; CBR, clinical benefit rate; PFS, progression-free survival; PPS, post-progression survival; OS, overall survival.

a: Pearson’s chi-square test using continuity correction.

### Factors influencing the overall efficacy

**Figure 3:**
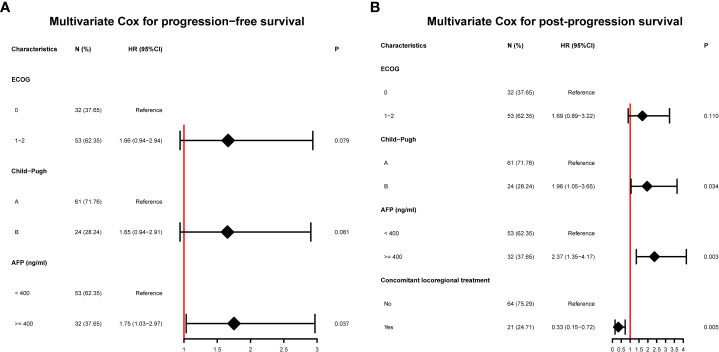
Risk factors for survival. **(A)** Multivariate Cox proportional hazard model for progression-free survival; **(B)** Multivariate Cox proportional hazard model for post-progression survival. ECOG, Eastern Cooperative Oncology Group; AFP, alpha-fetoprotein.

The multivariate Cox proportional hazards model suggested that AFP ≥ 400 ng/mL [1.75 (1.03-2.97), P = 0.037] was a risk factor for PFS. For OS, Child–Pugh B [1.96 (1.05-3.65), P = 0.034] and AFP ≥ 400 ng/mL [2.37 (1.35-4.17), P = 0.003] were risk factors, and concomitant locoregional therapy [0.33 (0.15-0.72), P =0.005] was a protective factor (**Table 4** and **Figure 3**).

**Table 4:** Risk factors for progression-free survival and post-progression survival.

Characteristics	Univariate Cox		Multivariate Cox	
	HR (95% CI)	P	HR (95% CI)	P
**Progression-free survival**				
Cohort: PLAGH vs. PUMCH	1.16 (0.66-2.02)	0.608		
Group: ICI+Others vs. ICI+Lenva	1.35 (0.77-2.37)	0.297		
Age: ≥ 65 vs. < 65	0.83 (0.42-1.64)	0.585		
Sex: Male vs. Female	0.73 (0.37-1.46)	0.375		
HBV infection: Yes vs. No	1.23 (0.61-2.51)	0.563		
HCV infection: Yes vs. No	0.69 (0.1-5.05)	0.719		
Alcohol consumption: Yes vs. No	1.02 (0.57-1.8)	0.956		
ECOG: 1-2 vs. 0	1.95 (1.13-3.37)	0.017	1.66 (0.94-2.94)	0.079
Child–Pugh: B vs. A	1.85 (1.08-3.18)	0.025	1.65 (0.94-2.91)	0.081
AFP (ng/ml): ≥ 400 vs. < 400	1.74 (1.03-2.94)	0.037	1.75 (1.03-2.97)	0.037
Tumor number: ≥ 2 vs. 1	1.54 (0.61-3.85)	0.359		
Macrovascular invasion: Yes vs. No	1.38 (0.83-2.29)	0.216		
Extrahepatic Metastasis: Yes vs. No	1.14 (0.67-1.92)	0.635		
BCLC staging: C vs. B	1.3 (0.52-3.26)	0.576		
Tumor size (cm): ≥ 5 vs. < 5	1.2 (0.69-2.08)	0.513		
Previous systemic treatment: Yes vs. No	0.92 (0.53-1.57)	0.753		
Previous locoregional treatment: Yes vs. No	2.08 (0.83-5.2)	0.118		
Concomitant locoregional treatment: Yes vs. No	0.84 (0.47-1.49)	0.553		
**Post-progression survival**				
Cohort: PLAGH vs. PUMCH	1.25 (0.67-2.32)	0.487		
Group: ICI+Others vs. ICI+Lenva	0.92 (0.49-1.7)	0.785		
Age: ≥ 65 vs. < 65	0.86 (0.42-1.76)	0.671		
Sex: Male vs. Female	1.54 (0.61-3.87)	0.362		
HBV infection: Yes vs. No	0.95 (0.5-1.82)	0.888		
HCV infection: Yes vs. No	2.17 (0.67-7.05)	0.197		
Alcohol consumption: Yes vs. No	0.98 (0.51-1.87)	0.949		
ECOG: 1-2 vs. 0	2.34 (1.28-4.27)	0.006	1.69 (0.89-3.22)	0.11
Child–Pugh: B vs. A	2.1 (1.17-3.75)	0.012	1.96 (1.05-3.65)	0.034
AFP (ng/ml): ≥ 400 vs. < 400	2.16 (1.24-3.76)	0.007	2.37 (1.35-4.17)	0.003
Tumor number: ≥ 2 vs. 1	1.9 (0.59-6.13)	0.28		
Macrovascular invasion: Yes vs. No	1.28 (0.74-2.22)	0.38		
Extrahepatic Metastasis: Yes vs. No	1.51 (0.87-2.62)	0.147		
BCLC staging: C vs. B	1.94 (0.7-5.4)	0.203		
Tumor size (cm): ≥ 5 vs. < 5	1.86 (0.99-3.51)	0.054		
Previous systemic treatment: Yes vs. No	1.06 (0.59-1.91)	0.833		
Previous locoregional treatment: Yes vs. No	1.46 (0.62-3.44)	0.382		
Concomitant locoregional treatment: Yes vs. No	0.35 (0.16-0.75)	0.007	0.33 (0.15-0.72)	0.005

PUMCH, Peking Union Medical College Hospital; PLAGH, Chinese Peoples’ Liberation Army General Hospital; HBV, hepatitis B virus; HCV, hepatitis C virus; NBNC, no HBV or HCV infection; AFP, alpha-fetoprotein; BCLC, Barcelona Clinic Liver Cancer.

### Adverse events

All patients were assessable for adverse events. AEs were recorded in all patients, and severe AEs (SAEs) were recorded in 46 (54.1%) patients. No AE-related deaths occurred. The most common AEs (>25%) were fatigue (45.9%), hyperbilirubinemia (38.8%), hypoalbuminemia (37.7%), elevated aspartate aminotransferase (36.5%), hypertension (35.3%) and decreased appetite (34.1%). For SAEs, hypertension (11.8%), diarrhea (8.2%), elevated aspartate aminotransferase (7.1%), fatigue (5.9%), abdominal pain (5.9%), rash (5.9%), anemia (5.9%) and gastrointestinal bleeding (5.9%) were the most common. The overall incidence of AEs in the ICI+Lenva group was slightly lower than that in the ICI+Others group (46.6% vs. 70.4%), with a marginally significant difference (P = 0.04) (**Table 5**).

**Table 5:** Adverse events.

Adverse Events	Overall		ICI+Lenva		ICI+Others	
	Any grade	Grade 3-4	Any grade	Grade 3-4	Any grade	Grade 3-4
Summary	85 (100)	46 (54.12)	58 (100)	27 (46.55)	27 (100)	19 (70.37)
Fatigue	39 (45.88)	5 (5.88)	24 (41.38)	1 (1.72)	15 (55.56)	4 (14.81)
Hyperbilirubinemia	33 (38.82)	4 (4.71)	23 (39.66)	2 (3.45)	10 (37.04)	2 (7.41)
Hypoalbuminemia	32 (37.65)	1 (1.18)	24 (41.38)	1 (1.72)	8 (29.63)	0 (0)
Aspartate aminotransferase increased	31 (36.47)	6 (7.06)	20 (34.48)	5 (8.62)	11 (40.74)	1 (3.7)
Hypertension	30 (35.29)	10 (11.76)	23 (39.66)	5 (8.62)	7 (25.93)	5 (18.52)
Decreased appetite	29 (34.12)	1 (1.18)	22 (37.93)	0 (0)	7 (25.93)	1 (3.7)
Hypothyroidism	21 (24.71)	0 (0)	16 (27.59)	0 (0)	5 (18.52)	0 (0)
Alanine aminotransferase increased	19 (22.35)	4 (4.71)	14 (24.14)	3 (5.17)	5 (18.52)	1 (3.7)
Diarrhea	18 (21.18)	7 (8.24)	15 (25.86)	6 (10.34)	3 (11.11)	1 (3.7)
Decreased platelet count	18 (21.18)	3 (3.53)	11 (18.97)	3 (5.17)	7 (25.93)	0 (0)
Abdominal pain	17 (20)	5 (5.88)	12 (20.69)	4 (6.9)	5 (18.52)	1 (3.7)
Rash	16 (18.82)	5 (5.88)	12 (20.69)	3 (5.17)	4 (14.81)	2 (7.41)
Anemia	15 (17.65)	5 (5.88)	10 (17.24)	2 (3.45)	5 (18.52)	3 (11.11)
Electrolyte disturbance	15 (17.65)	2 (2.35)	10 (17.24)	2 (3.45)	5 (18.52)	0 (0)
Gastrointestinal bleeding	14 (16.47)	5 (5.88)	8 (13.79)	3 (5.17)	6 (22.22)	2 (7.41)
Proteinuria	14 (16.47)	3 (3.53)	12 (20.69)	3 (5.17)	2 (7.41)	0 (0)
Ascites	13 (15.29)	1 (1.18)	11 (18.97)	1 (1.72)	2 (7.41)	0 (0)
Decreased white blood cell count	13 (15.29)	1 (1.18)	10 (17.24)	1 (1.72)	3 (11.11)	0 (0)
Fever	10 (11.76)	2 (2.35)	5 (8.62)	1 (1.72)	5 (18.52)	1 (3.7)
Nausea	8 (9.41)	1 (1.18)	5 (8.62)	1 (1.72)	3 (11.11)	0 (0)
Abdominal distension	7 (8.24)	0 (0)	5 (8.62)	0 (0)	2 (7.41)	0 (0)
Edema	7 (8.24)	0 (0)	6 (10.34)	0 (0)	1 (3.7)	0 (0)
Gingival bleeding	7 (8.24)	0 (0)	4 (6.9)	0 (0)	3 (11.11)	0 (0)
Vomiting	7 (8.24)	2 (2.35)	6 (10.34)	2 (3.45)	1 (3.7)	0 (0)
Decreased weight	7 (8.24)	0 (0)	6 (10.34)	0 (0)	1 (3.7)	0 (0)
Hand-foot syndrome	6 (7.06)	2 (2.35)	6 (10.34)	2 (3.45)	5 (18.5)	2 (7.41)
Pneumonia	3 (3.53)	3 (3.53)	1 (1.72)	1 (1.72)	2 (7.41)	2 (7.41)
Peptic ulcer	1 (1.18)	0 (0)	1 (1.72)	0 (0)	0 (0)	0 (0)

## Discussion

Our study evaluated the clinical outcomes of MTAs plus ICIs after the progression of lenvatinib. In this cohort of 85 patients from two medical centers in China, we observed an ORR of 10.6%, with a median PFS of 4.5 months, a median PPS of 14.0 months, and a median OS of 22.1 months. No AE-related deaths occurred. This study indicated that later-line MTA+ICI treatment is generally safe and effective after the progression of lenvatinib treatment.

Lenvatinib demonstrated promising antitumor activity in the first-line treatment of aHCC (2), and it performed well in second-line and later-line studies in the real world (21, 22). However, tumor resistance is unavoidable. In the REFLECT study, patients in the lenvatinib arm who progressed without subsequent therapy had a median OS of approximately 11.5 months, which was not significantly different from 9.1 months with first-line sorafenib without subsequent therapies (HR 0.90, 95% CI: 0.75-1.09) (2). Sequential immunotherapy after the progression of MTAs is a promising treatment option. At present, the efficacy and safety of sequential MTAs and immunotherapy after the progression of sorafenib have been reported in prospective studies (6, 9, 11). Since lenvatinib was approved for HCC treatment not long ago, data on sequential therapy after lenvatinib progression are limited. The efficacy and safety of regorafenib and/or regorafenib plus PD-1 inhibitors after the progression of first-line sorafenib or lenvatinib treatment in real-world situations have been reported recently (16). However, considering the complicated lenvatinib lines in the real world and various factors affecting subsequent treatment decisions, the optimal sequential MTA+ICI regimen after the progression of lenvatinib and whether it needs to be actively switched to MTAs remain unclear. Our real-world research answered this question for the first time.

Later-line systemic therapy is likely to bring prognostic benefits. A *post hoc* analysis of the REFLECT study by Alsina et al. showed that after the progression of first-line lenvatinib, 32.6% of patients received subsequent systemic therapy. Most subsequent treatments were targeted agents, while only 3.1% of patients were treated with immunotherapy. Second-line systemic therapy was associated with prolonged survival (median OS: 20.8 vs. 11.5 months) (15). In the RESORCE study, the median OS of Child–Pugh A patients receiving regorafenib as a second-line MTA was 10.6 months (11). The KEYNOTE-240 study finally reported a median OS of 13.9 months and a median PFS of 3.0 months with pembrolizumab monotherapy after sorafenib progression (6). Furthermore, systemic therapy following the progression of combination therapy was associated with a better prognosis in our study (**Figure S1**).

It seemed that the MTA plus ICI pattern in our study performed better than MTA or ICI monotherapy. The median PPS after the progression of lenvatinib treatment in our cohort reached 14.0 months, which was higher than that in RESORCE and KEYNOTE-240 (6, 11). Moreover, the median OS reached 22.2 months, exceeding the median OS of patients in the lenvatinib arm who received later-line therapy in the REFLECT study. The comparison preliminarily displayed the effectiveness of subsequent MTA plus ICI after the progression of lenvatinib treatment. Huang et al. explored the efficacy of regorafenib and regorafenib plus PD-1 inhibitors after the progression of first-line sorafenib or lenvatinib treatment. The regorafenib plus PD-1 inhibitor group had better OS (median 13.4 vs. 9.9 months; *P* = 0.023) (16). In this research, we further analyzed the cohort. After extracting the subgroup of patients with first-line lenvatinib treatment, we found that the efficacy in the subgroup (median PFS: 4.6 months, median PPS: 14.0 months, and median OS: 22.1 months) was similar to the efficacy in the overall cohort (median PFS: 4.5 months, median PPS: 14.0 months, and median OS: 22.1 months). Moreover, PPS in both the subgroup and overall cohort was similar or slightly better than this endpoint reported in Huang’s cohort. The comprehensive analysis of these two studies indicated that later-line MTA+ICI was associated with a better prognosis, regardless of the progression of lines of lenvatinib treatment.

Combination therapy can achieve the “1+1>2” effect; however, the mechanism of combined synergism is not yet fully understood but might be related to the tumor microenvironment. Recent studies have shown that protein kinase Cα is directly phosphorylated at S226, activating transcription, inducing macrophage recruitment and M2-like polarization, and driving immune escape and resistance to ICIs. Lenvatinib can restore sensitivity to ICIs by blocking the protein kinase Cα/zinc finger protein 64/macrophage colony-stimulating factor axis, thus remodeling the tumor microenvironment (23). In addition, VEGFR-2 inhibition was associated with upregulation of IFN-γ and PD-L1 expression, and simultaneous blockade of PD-1 and VEGFR-2 in HCC can promote vascular normalization and enhance antitumor immune responses (24). With the development of tumor immunology, it will be possible to further expand the patient population suitable for immunotherapy by combining different MTAs or local therapy.

In the real world, many patients continued lenvatinib treatment after the progression of front-line lenvatinib treatment with the help of Patient Assistance Programs, lacking enough support to select another MTA. Unfortunately, no clinical research has discussed the necessity of switching to another MTA combined with immunotherapy as a late-line treatment. In our study, the ICI+Lenva and ICI+Others groups had similar follow-up periods and comparable baseline characteristics, and no significant differences were observed between these two groups in the short-term and long-term efficacy endpoints. In addition, lenvatinib performed well in a cost-effectiveness analysis (25–29), so the rechallenge of lenvatinib plus ICI is likely to be a better choice. Future prospective clinical trials are needed to prove the above hypothesis.

Physical performance, liver function, and AFP levels were risk factors for short- and long-term outcomes in our study, which have been thoroughly discussed in previous articles. At the same time, concomitant interventional therapy was a protective factor for long-term efficacy. Previous studies also reported the synergistic effect of interventional therapy on immunotherapy (30, 31). Our study further confirmed that interventional therapy improved the prognosis of patients treated with ICI plus MTA following lenvatinib. The safety of this MTA plus ICI pattern was acceptable. The AE spectrum was similar to that in REFLECT, RESORCE, KEYNOTE-240, and IMbrave150 (2, 6, 11, 32), and no fatal AEs occurred.

Several limitations existed in our study. Unlike global multicenter randomized controlled trials, this study is a retrospective real-world observational study including only 85 patients from two medical centers, which could not fully represent Chinese HCC patients. In addition, multiple ICIs and MTAs were used in this study, and complex treatment regimens may impact survival outcomes. At present, many first-line and second-line treatments for HCC have been approved. In the future, more complete randomized controlled trials are needed to explore the treatment options following progression after lenvatinib.

## Conclusion

In conclusion, this study provides valuable evidence that ICI plus MTA in HCC patients after the progression of lenvatinib presented potential antitumor activity and safety. Furthermore, concomitant locoregional treatment seemed to be associated with better OS.

## Data availability statement

The research data are not publicly available on ethical grounds. However, inquiries regarding all data analyzed in this study can be directed to the corresponding author.

## Ethics statement

The studies involving human participants were reviewed and approved by Ethics Committees of Peking Union Medical College Hospital Ethics Committees of The Fifth Medical Centre of PLA General Hospital. Written informed consent for participation was not required for this study in accordance with the national legislation and the institutional requirements.

## Author contributions

Conceptualization, HZ and YLu. Methodology, FX, BC, XuY and HW. Software, GZ and YaW. Validation, YcW and NZ. Formal analysis, JX. Investigation, JL. Resources, YL, HS and ZX. Data curation, KL, XC, YS and XbY. Writing—original draft prep-aration, FX, BC, XuY and HW. Writing—review and editing, FX, BC, XuY and HW. Visualization, ZL. Supervision, ZL, YM, XS, YLu and HZ. Project administration, HZ. Funding acquisition, XBY, XS and HZ. All authors contributed to the article and approved the submitted version.
