# Supplementary material for: Efficacy of immune checkpoint inhibitors plus molecular targeted agents after the progression of lenvatinib for advanced hepatocellular carcinoma
Source: Front Immunol. 2022 Dec 9;13:1052937. doi: 10.3389/fimmu.2022.1052937 (PMC9780480; doi:10.3389/fimmu.2022.1052937)
Supplement: Supplementary file 1 [file DataSheet_1.docx]

# Efficacy of immune checkpoint inhibitors plus molecular targeted agents after the progression of lenvatinib for advanced hepatocellular carcinoma

# Supplementary Material

## Table S1. Reasons for discontinuation of combination therapy directly after lenvatninb treatment.

|  | **Patients discontinued treatment (n = 61)** |
| --- | --- |
| Progression of disease (including death) | 54 |
| Adverse events | 7 |

## Table S2. Post-progression treatment after combination therapy.

|  | **Patients (n = 19)** |
| --- | --- |
| ICI plus TKI | 9 |
| TKI | 10 |

## Figure S1. Survival analysis plot of patients with or without systemic treatment after progression of ICI+MTA.


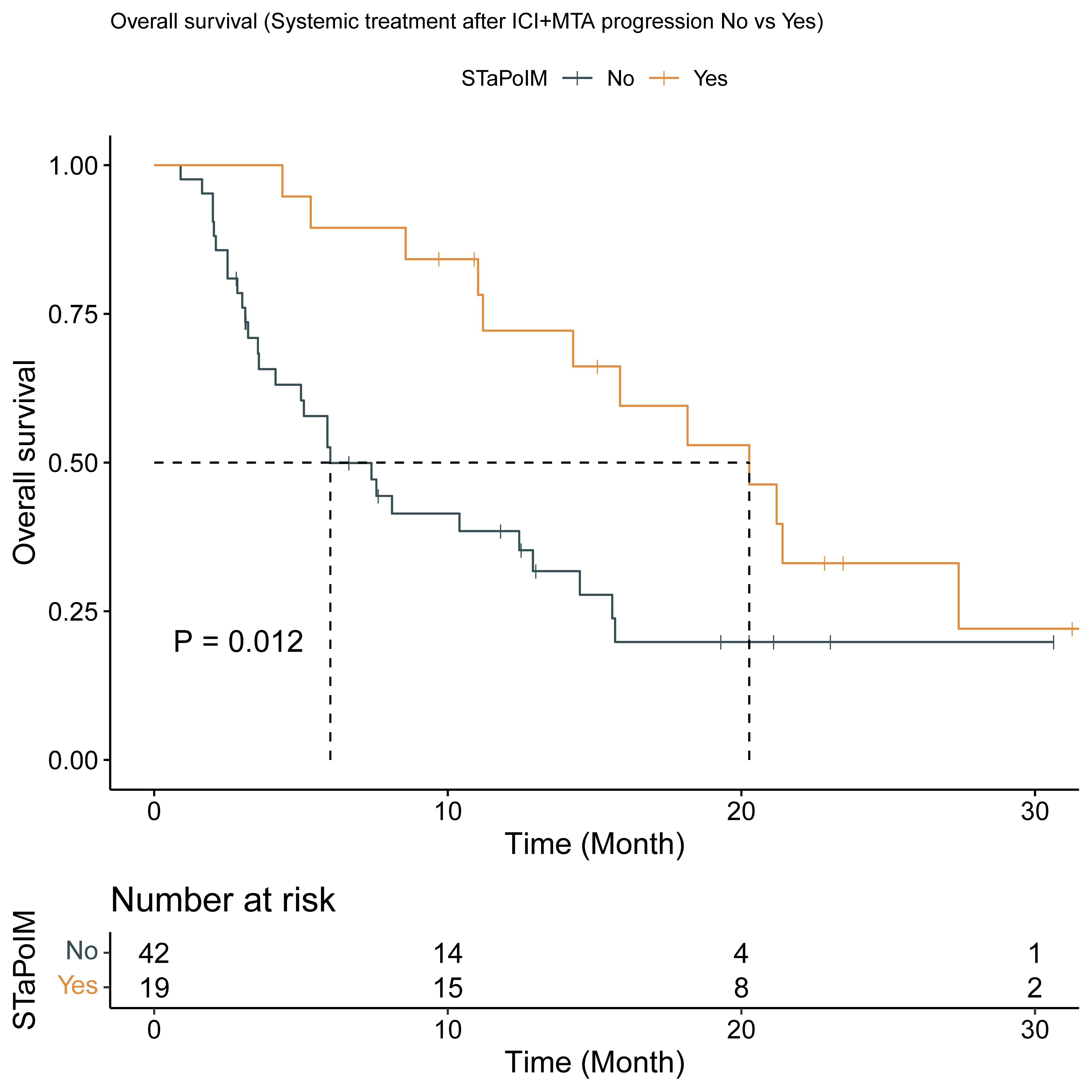
 STaPoIM, Systemic Treatment after Progression of ICI+MTA
